# Both α_1_- and α_2_-adrenoceptors in the Insular Cortex Are Involved in the Cardiovascular Responses to Acute Restraint Stress in Rats

**DOI:** 10.1371/journal.pone.0083900

**Published:** 2014-01-03

**Authors:** Fernando H. F. Alves, Carlos C. Crestani, Leonardo B. M. Resstel, Fernando M. A. Corrêa

**Affiliations:** 1 Department of Pharmacology, School of Medicine of Ribeirão Preto, University of São Paulo, Ribeirão Preto, SP, Brazil; 2 Department of Natural Active Principles and Toxicology, School of Pharmaceutical Sciences of Araraquara, Univ. Estudual Paulista - UNESP, Araraquara, SP, Brazil; University of Torino, Italy

## Abstract

The insular cortex (IC) is a limbic structure involved in cardiovascular responses observed during aversive threats. However, the specific neurotransmitter mediating IC control of cardiovascular adjustments to stress is yet unknown. Therefore, in the present study we investigated the role of local IC adrenoceptors in the cardiovascular responses elicited by acute restraint stress in rats. Bilateral microinjection of different doses (0.3, 5, 10 and 15 nmol/100 nl) of the selective α_1_-adrenoceptor antagonist WB4101 into the IC reduced both the arterial pressure and heart rate increases elicited by restraint stress. However, local IC treatment with different doses (0.3, 5, 10 and 15 nmol/100 nl) of the selective α_2_-adrenoceptor antagonist RX821002 reduced restraint-evoked tachycardia without affecting the pressor response. The present findings are the first direct evidence showing the involvement of IC adrenoceptors in cardiovascular adjustments observed during aversive threats. Our findings indicate that IC noradrenergic neurotransmission acting through activation of both α_1_- and α_2_-adrenoceptors has a facilitatory influence on pressor response to acute restraint stress. Moreover, IC α_1_-adrenoceptors also play a facilitatory role on restraint-evoked tachycardiac response.

## Introduction

Stress situations happen during real or perceived threat to homeostasis or well-being. Stressors include either interoceptive changes (e.g., blood volume or osmolality changes) or environmental threat that may be physical (e.g., hypoxia) or psychological (e.g., presence of a predator). During stress a spectrum of physiological responses are evoked to maintain the physiologic integrity of the organism [1]. The physiological responses to stress are mainly characterized by autonomic nervous system alterations, increase in plasma catecholamine levels and activation of the hypothalamus-pituitary-adrenal (HPA) axis [1], [2]. Autonomic responses include increase on both blood pressure and heart rate (HR) [3], [4]. Furthermore, cardiovascular changes during stress are accompanied by a resetting of baroreflex toward higher arterial pressure values, thus allowing simultaneous blood pressure and HR increases [5]–[8].

Several central nervous system areas, including the prefrontal cortex, were described to be part of the brain circuitry involved on cardiovascular adjustments during stress [3], [4], [9], [10]. In rats, two regions of the prefrontal cortex involved in control of cardiovascular function are the insular cortex (IC) and the medial prefrontal cortex (MPFC) [11], [12]. It has been described that the IC is involved in cardiovascular control [13]–[15] and baroreflex modulation [16]–[19]. Furthermore, previous results from our group demonstrated that bilateral microinjection of the unspecific neurotransmitter blocker CoCl_2_ into the IC of rats reduced both cardiovascular and behavioral responses evoked by either conditioned (contextual fear conditioning) or unconditioned (acute restraint stress) aversive stimuli [2], [20]. These results provided the first evidence of a role of the IC in cardiovascular adjustments during stress. However, due to the nonselective blockade of local neurotransmission caused by CoCl_2_ [21], [22], the specific neurotransmitter involved in the IC modulation of cardiovascular responses to stress is yet unknown.

Central noradrenergic circuitry is shortly activated after a stressful event [1]. Conversely, it has been identified an enhanced release of noradrenaline after stress in several limbic brain regions including the central (CeA) and medial (MeA) amygdaloid nuclei, bed nucleus of the stria terminalis (BNST), lateral septal area (LSA), hippocampus and prefrontal cortex [1], [23]–[26]. Noradrenergic terminals in the prefrontal cortex originate mainly from the locus coeruleus and play an important role in the regulation of cortical function [27]–[30]. We have previously reported that noradrenergic neurotransmission within the IC is involved in the modulation of baroreflex activity [31]. Also, microinjection of noradrenaline into the IC causes elevation of blood pressure and bradycardia [14]. Although above evidence, the involvement of IC noradrenergic neurotransmission in the control of cardiovascular function during stress situations has never been investigated.

Therefore, given the involvement of IC-noradrenergic neurotransmission in cardiovascular control, we hypothesized an involvement of IC α-adrenoceptors in cardiovascular responses elicited by acute restraint stress in rats. To test this hypothesis, we investigated the effect of bilateral microinjections into the IC of selective α-adrenoceptor antagonists in restraint-evoked pressor and tachycardiac responses.

## Experimental procedure

### Ethical approval and animals

Experimental procedures were carried out following protocols approved by the Ethical Review Committee of the School of Medicine of Ribeirão Preto, (process number: 167/2007), which complies with the guiding principles for research involving animals and human beings of the National Institutes of Health. Fifty-seven male Wistar rats weighing approximately 250 g were used in the present experiment. Rats were housed in plastic cages in a temperature-controlled room (25°C) at the Animal Care Unit of the Department of Pharmacology, School of Medicine of Ribeirão Preto. Rats were kept under a 12 h :12 h light–dark cycle (lights on between 06:00 am and 6:00 pm) and had free access to water and standard laboratory food, except during the experimental period.

### Surgical preparation

Five days before the experiment, the rats were anesthetized with tribromoethanol (250 mg/kg, i.p.). After local anesthesia with 2% lidocaine, the skull was surgically exposed and stainless steel guide cannulas (26 G) were implanted bilaterally in the IC, using a stereotaxic apparatus (Stoelting, Wood Dale, Illinois, USA). Stereotaxic coordinates for cannula implantation in the IC were selected from the rat brain atlas of Paxinos and Watson (1997) and were: antero-posterior = +11.7 mm from interaural, lateral = 4.0 mm from the medial suture and dorso-ventral = −4.5 mm from the skull. Cannulas were fixed to the skull with dental cement and one metal screw. After surgery, the animals were treated with a polyantibiotic preparation of streptomycins and penicillins (i.m., 0.27 mg/kg, Pentabiotico, Fort Dodge®, Campinas, SP, Brazil) to prevent infection, and with the non-steroidal anti-inflammatory flunixine meglumine (2.5 mg/kg, i.m.; Banamine®, Schering Plough, Cotia, SP, Brazil) for post-operative analgesia.

One day before the experiment, rats were anesthetized with tribromoethanol (250 mg/kg, i.p.) and a catheter (a 4 cm segment of PE-10 heat-bound to a 13 cm segment of PE-50, Clay Adams, Parsippany, NJ, USA) was inserted into the abdominal aorta through the femoral artery, and later on used for arterial pressure and HR recording. The catheters were tunneled under the skin and exteriorized on the animal's dorsum. After surgery, the animals were treated with the non-steroidal anti-inflammatory flunixine meglumine (2.5 mg/kg, i.m.) for post-operative analgesia.

### Measurement of Cardiovascular Responses

On the day of the experiment, the arterial cannula was connected to a pressure transducer and pulsatile arterial pressure was recorded using an HP-7754A amplifier (Hewlett Packard, Palo Alto, CA, USA) and an acquisition board (Biopac M-100, Goleta, CA, USA) connected to a personal computer. Mean arterial pressure (MAP) and HR values were derived from pulsatile arterial pressure recordings and were processed online.

### Drugs and solutions

WB4101 (Tocris, Westwoods Business Park Ellisville, MO, USA) and RX821002 (Tocris) were dissolved in artificial cerebrospinal fluid (ACSF) (ACSF composition: 100 mM NaCl; 2 mM Na_3_PO_4_; 2.5 mM KCl; 1 mM MgCl_2_; 27 mM NaHCO_3_; 2.5 mM CaCl_2_; pH = 7.4). Urethane (Sigma, St. Louis, MO, USA) and tribromoethanol (Sigma) were dissolved in saline (0.9% NaCl). Flunixine meglumine (Banamine®, Schering Plough, Brazil) and poly-antibiotic preparation of streptomycins and penicillins (Pentabiotico®, Fort Dodge, Brazil) were used as provided.

### Drug injection into the insular cortex

The needles (33 G, Small Parts, Miami Lakes, FL, USA) used for microinjection into the IC were 1 mm longer than the guide cannulas and were connected to a hand-driven 2 µl syringe (7002-KH, Hamilton Co., Reno, NV, USA) through a PE-10 tubing. Needles were carefully inserted into the guide cannulas without restraint the animals. After a 30 s period, the needle was removed and inserted into the second guide cannula for microinjection into the contralateral IC. Drugs were injected in a final volume of 100 nl [2], [20].

### Experimental procedure: acute restraint stress

On the trial day, animals were brought to the experimental room in their home cages. Animals were allowed one hour to adapt to the conditions of the experimental room, such as sound and illumination, before starting cardiovascular recordings. The experimental room was temperature controlled (25°C) and the room was acoustically isolated from the other rooms. Constant background noise was generated by an air extractor to minimize sound interference within the experimental room. Baseline values of MAP and HR were recorded for at least 30 min. In the sequence, independent groups of animals received bilateral microinjection into the IC of vehicle (ACSF, 100 nl) or different doses of either the selective α_1_-adrenoceptor antagonist WB4101 (0.3, 5, 10 or 15 nmol/100 nl) or the selective α_2_-adrenoceptor antagonist RX821002 (0.3, 5, 10 or 15 nmol/100 nl) [14], [31], [32]. Ten minutes later, rats were submitted to acute restraint stress by placing them into a plastic cylindrical restraint tube (diameter  = 6.5 cm, length  = 15 cm), which were ventilated by holes (1 cm of diameter) that comprised approximately 20% of the tube surface. The restraint session lasted 60 min, after which the rats were returned to their home cages [20], [33]. Each rat was submitted to one session of restraint in order to avoid habituation. Experiments were performed during the morning period in order to minimize possible circadian rhythm interferences.

### Histological determination of the microinjection sites

At the end of experiments, animals were anesthetized with urethane (1.25 g/kg, i.p.) and 100 nL of 1% Evan's blue dye was injected into the IC as a marker of the injection site. They were then submitted to intracardiac perfusion with 0.9% NaCl followed by 10% formalin. Brains were removed and post fixed for 48 h at 4°C and serial 40 µm-thick sections were cut using a cryostat (CM1900, Leica, Wetzlar, Germany). Sections were stained with 1% neutral red for light microscopy analysis. The placement of the microinjection needles was determined analyzing serial sections and identified according to the rat brain atlas of Paxinos and Watson (1997).

### Statistical Analysis

Statistical analysis was performed using Prism software (GraphPad, SanDiego, CA, USA). The results are presented as mean±S.E.M. The Student's t-test was used to compare basal values of MAP and HR before and after pharmacological treatments. Time-course curves of MAP and HR changes were compared using two-way ANOVA for repeated measurements (treatment vs time) with repeated measures on the second factor. When interactions between the factors were observed, one-way ANOVA followed by Bonferroni's post-hoc test was used to compare the effect of the treatments. Nonlinear regression analysis was performed to investigate the dose–effect relationship of treatment with crescents doses of WB4101 and RX821002 on cardiovascular responses to restraint stress.

## Results

**Figure 1:**
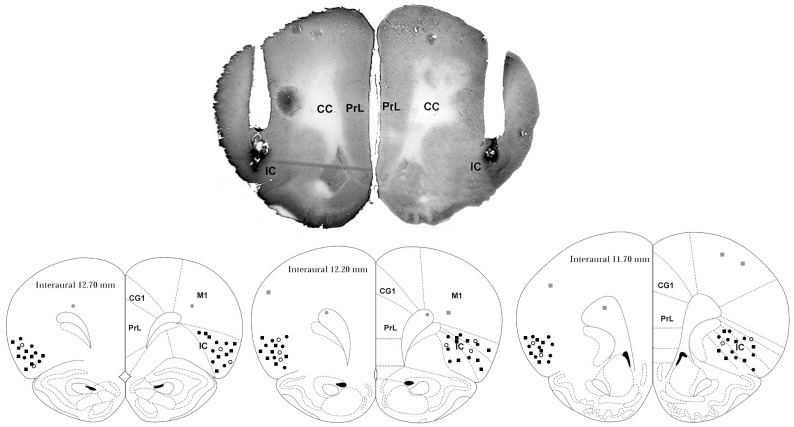
A photomicrograph of a coronal brain section, from one representative rat, which shows bilateral injection sites in the insular cortex. Diagrammatic representation based on the rat brain atlas of Paxinos and Watson (1997) indicating the microinjection sites of vehicle (white circles), WB4101 (black circles) and RX821002 (black squares) into the IC as well as WB4101 (gray circles) and RX821002 (gray squares) into structures surrounding the IC. Cg1 – cingulate cortex, area; PrL – prelimbic cortex, M1 – primary motor cortex; insular cortex – insular cortex, cc – corpus callosum, forceps minor of the corpus callosum (fmi).

A representative photomicrograph of a coronal brain section depicting bilateral microinjection sites in the IC of one representative rat is presented in Figure 1. A diagrammatic representation showing microinjection sites of vehicle, WB4101 and RX821002 into the IC and WB4101 and RX821002 into structures surrounding the IC is also presented in Figure 1.

### Effect of IC pretreatment with different doses of the selective α_1_-adrenoceptor antagonist WB4101 on cardiovascular changes evoked by acute restraint stress

**Figure 2:**
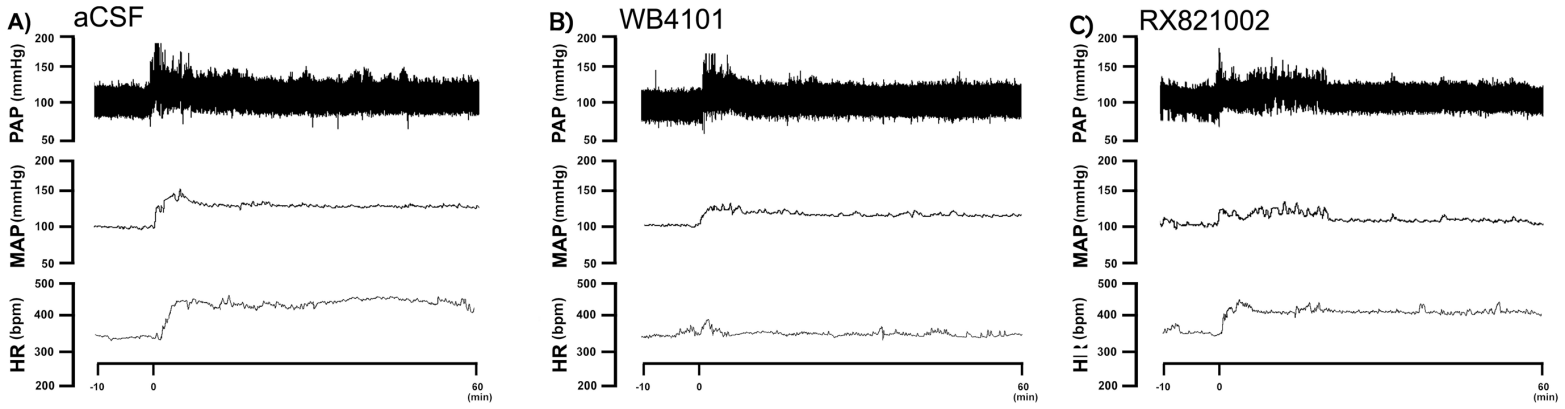
Representative recordings of mean arterial pressure (MAP), pulsatile arterial pressure (PAP) and heart rate (HR) of representative rats submitted to acute restraint stress. **A)** Recordings of MAP, PAP and HR of one representative rat treated with vehicle (ACSF) into the insular cortex and submitted to acute restraint stress. The onset of restraint is at t = 0. **B)** Recordings of MAP, PAP and HR of one representative rat treated with the selective α_1_-adrenoceptor antagonist WB4101 into the insular cortex and submitted to acute restraint stress. The onset of restraint is at t = 0. Note the decrease in the MAP, PAP and HR responses to restraint stress in animals treated with WB4101 into the insular cortex. C) Recordings of MAP, PAP and HR of one representative rat treated with the selective α_2_-adrenoceptor antagonist RX821002 into the insular cortex and submitted to acute restraint stress. The onset of restraint is at t = 0. Note the decrease in the MAP and PAP responses to restraint stress in animals treated with RX821002 into the insular cortex.

**Figure 3:**
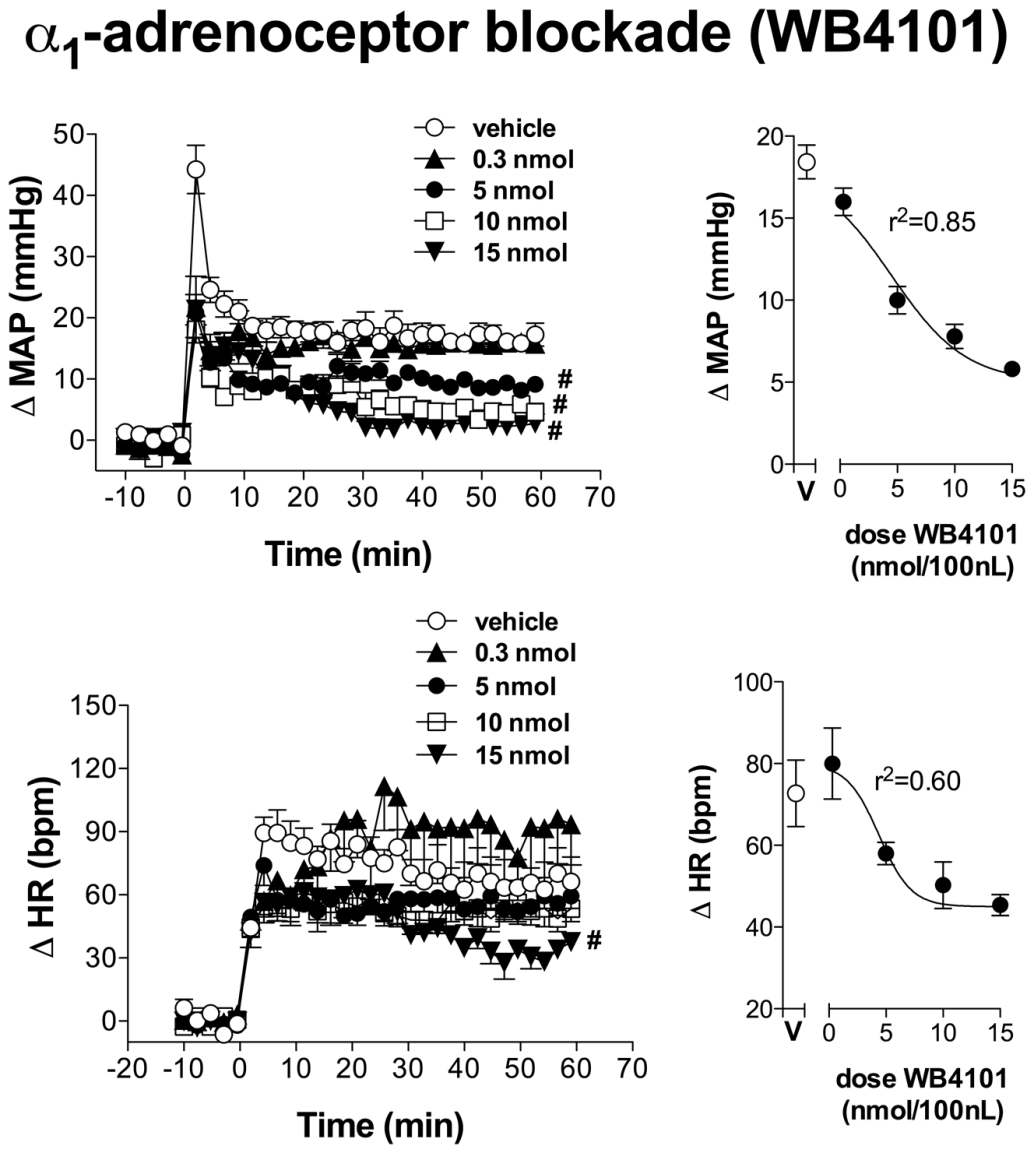
Changes in mean arterial pressure (ΔMAP) and heart rate (ΔHR) evoked by acute restraint stress in animals treated with different doses of the selective α_1_-adrenoceptor antagonist WB4101 into the insular cortex. **(Left)** Time-course of ΔMAP and ΔHR during acute restraint stress in rats treated with vehicle (ACSF, 100 nl, n = 7) or different doses (0.3, 5, 10 and 15 nmol/100 nl, n = 5/group) of WB4101 into the insular cortex. The onset of exercise was at t = 0. Circles represent the mean and bars the S.E.M. # P<0.05, indicates a significant difference over the whole restraint stress period compared to vehicle treated animals; ANOVA followed by Bonferroni's post test. **(Right)** ΔMAP and ΔHR during acute restraint stress in rats treated with increasing doses of WB4101 (0.3, 5, 10 and 15 nmol/100 nl). V: vehicle (ACSF, 100 nl). Dose–effect curves were generated by nonlinear regression analysis. Data shown represent the means±S.E.M. of the variation of MAP and HR during the 60 min of restraint. # P<0.05, significantly different from vehicle group; one-way ANOVA followed by Bonferroni's post test.

Bilateral microinjection of different doses (0.3, 5, 10 and 15 nmol/100 nL, n = 5/group) of the selective α_1_-adrenoceptor antagonist WB4101 into the IC did not affect either MAP or HR baseline values (Table 1). Representative experimental recordings showing effects of local microinjection of WB4101 into the IC on cardiovascular responses elicited by acute restraint stress are presented in Figure 2. Time-course analysis of restraint-evoked cardiovascular responses indicated that IC treatment with WB4101 (at doses of 5, 10 and 15 nmol/100 nl for MAP response and at dose of 15 nmol/100 nl for HR response) reduced both MAP (F_(4,660)_ = 336, P<0.0001) and HR (F_(4,660)_ = 50, P<0.0001) responses, when compared with ACSF-treated animals (n = 7) (Figure 3). There was also a significant effect over time for MAP (F_(29,660)_ = 70, P<0.0001) and HR (F_(29,660)_ = 27, P<0.0001) responses, as well as a treatment x time interaction for the pressor response (MAP: F_(116,660)_ = 6, P<0.0001; HR: F_(116,660)_ = 1, P>0.05). Nonlinear regression analysis revealed that WB4101 effects on restraint-evoked cardiovascular responses were dose-dependent, showing a significant association between drug dose and MAP (df = 18, r^2^ = 0.85, P<0.05) and HR (df = 13, r^2^ = 0.60, P<0.05) increases (Figure 3). The injection of WB4101 (n = 2) into structures surrounding the IC did not affect both MAP (F_(1,60)_ = 2,9, P>0.05) and HR (F_(1,60)_ = 0,2, P>0.05) responses to restraint stress.

**Table 1:** Effect of bilateral microinjections into the IC of crescent doses (0.3, 5, 10 and 15 nmol/100 nl) of the selective α_1_-adrenoceptor antagonist WB4101 on mean arterial pressure (MAP) and heart rate (HR) baseline.

Treatment	WB4101 0.3 nmol	WB4101 5 nmol	WB4101 10 nmol	WB4101 15 nmol
N	5	5	5	5
MAP–Before treat.	102±3	98±3	96±3	103±3
MAP–After treat.	100±4	100±4	104±3	98±6
Statistic	*t* = 0.4, *P*>0.05	*t* = 0.3, *P*>0.05	*t* = 2.6, *P*>0.05	*t* = 1.8, *P*>0.05
HR – Before treat.	368±12	364±10	356±9	356±7
HR – After treat.	356±13	382±9	377±12	367±10
Statistic	*t* = 0.4, *P*>0.05	*t* = 1, *P*>0.05	*t* = 2.5, *P*>0.05	*t* = 1.8, *P*>0.05

Student's *t*-test.

### Effect of IC pretreatment with different doses of the selective α_2_-adrenoceptor antagonist RX821002 on cardiovascular changes evoked by acute restraint stress

**Figure 4:**
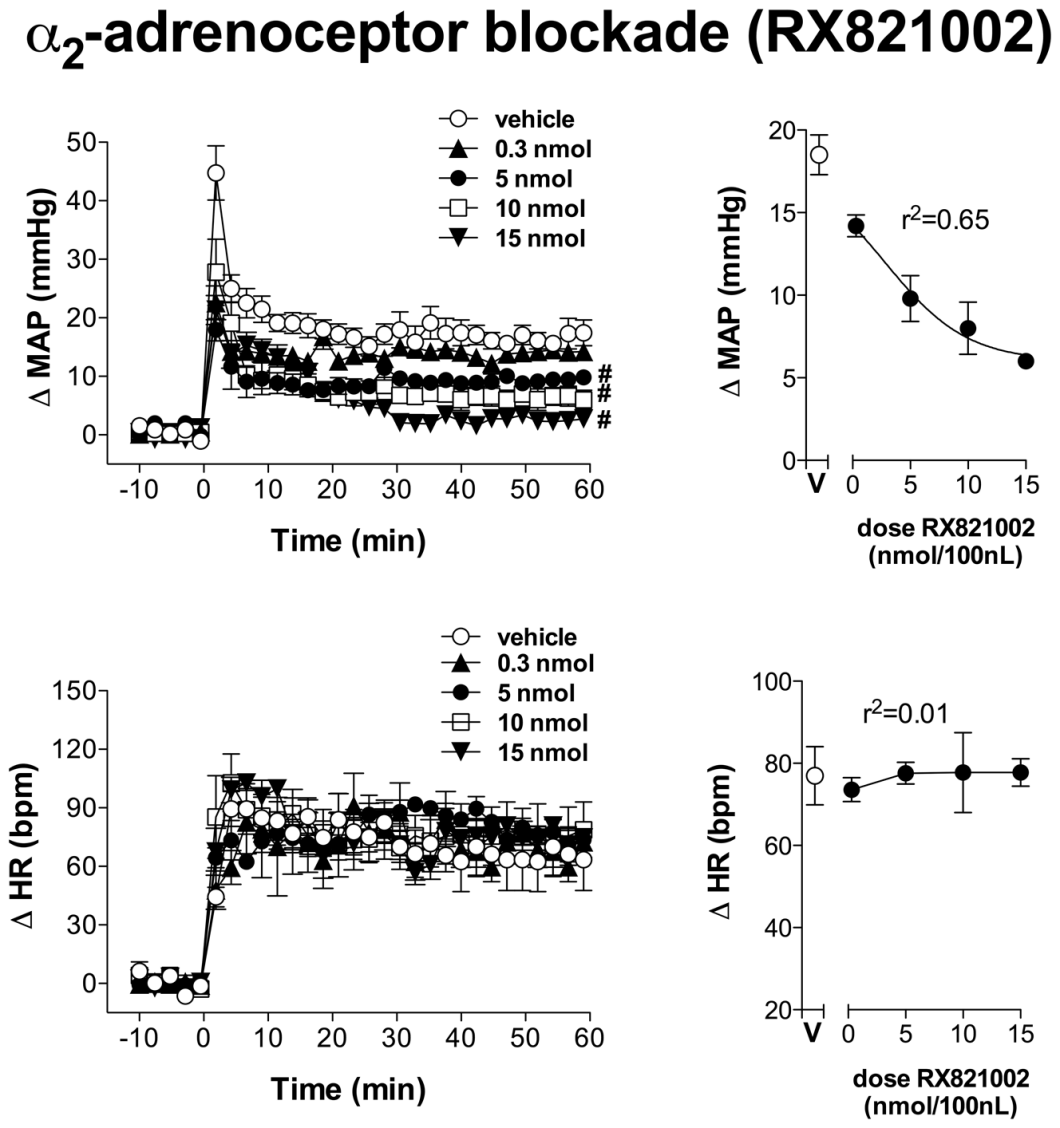
Changes in mean arterial pressure (ΔMAP) and heart rate (ΔHR) evoked by acute restraint stress in animals treated with different doses of the selective α_2_-adrenoceptor antagonist RX821002 into the insular cortex. (Left) Time-course of ΔMAP and ΔHR during acute restraint stress in rats treated with vehicle (ACSF, 100 nl, n = 6) or different doses (0.3, 5, 10 and 15 nmol/100 nl, n = 5/group) of the selective α_2_-adrenoceptor antagonist RX821002 into the insular cortex. The onset of exercise was at t = 0. Circles represent the mean and bars the S.E.M. # P<0.05, indicates a significant difference over the whole restraint stress period compared to vehicle treated animals; ANOVA followed by Bonferroni's post test. (Right) ΔMAP and ΔHR during restraint stress in rats treated with increasing doses of RX821002 (0.3, 5, 10 and 15 nmol/100 nl). V: vehicle (ACSF, 100 nl). Dose–effect curves were generated by nonlinear regression analysis. Data shown represent the means±S.E.M. of the variation of MAP and HR during the 60 min of restraint. # P<0.05, significantly different from vehicle group; one-way ANOVA followed by Bonferroni's post test.

Bilateral microinjection of different doses (0.3, 5, 10 and 15 nmol/100 nL, n = 5/group) of the selective α_2_-adrenoceptor antagonist RX821002 into the IC did not affect either MAP or HR baseline values (Table 2). Representative experimental recordings showing effects of RX821002 microinjection into the IC on cardiovascular responses induced by acute restraint stress are presented in Figure 2. Time-course analysis of restraint-evoked cardiovascular responses indicated that IC treatment with RX821002 at doses of 5, 10 and 15 nmol/100 nl reduced MAP response (F_(4,630)_ = 216, P<0.0001) without affecting restraint-evoked tachycardia (F_(4,630)_ = 2, P>0.05), when compared with ACSF-treated animals (n = 6) (Figure 4). There was also a significant effect over time for MAP (F_(29,630)_ = 54, P<0.0001) and HR (F_(29,630)_ = 42, P<0.0001) responses, as well as a treatment x time interaction for the pressor response (MAP: F_(116,630)_ = 3, P<0.0001; HR: F_(116,630)_ = 0.6, P>0.05). Nonlinear regression analysis revealed that RX821002 effect on restraint-evoked pressor response was dose-dependent, showing a significant association between drug doses and MAP increase (df = 18, r^2^ = 0.65, P<0.05) (Figure 4). The injection of RX821102 into structures surrounding the IC did not affect both MAP (F_(1,60)_ = 1,4, P>0.05) and HR (F_(1,60)_ = 0,005, P>0.05) responses to restraint stress.

**Table 2:** Effect of bilateral microinjections into the IC of crescent doses (0.3, 5, 10 and 15 nmol/100 nl) of the selective α_2_-adrenoceptor antagonist RX821002 on mean arterial pressure (MAP) and heart rate (HR) baseline.

Treatment	RX821002 0.3 nmol	RX821002 5 nmol	RX821002 10 nmol	RX821002 15 nmol
N	5	5	5	5
MAP–Before treat.	96±3	99±3	103±4	95±2
MAP–After treat.	106±4	101±2	102±5	101±2
Statistic	*t* = 2.6, *P*>0.05	*t* = 0.5, *P*>0.05	*t* = 0.3, *P*>0.05	*t* = 1.3, *P*>0.05
HR – Before treat.	356±9	353±10	356±7	384±12
HR – After treat.	380±16	362±14	358±12	362±11
Statistic	*t* = 1.5, *P*>0.05	*t* = 0.8 *P*>0.05	*t* = 0.1, *P*>0.05	*t* = 1, *P*>0.05

Student's *t*-test.

## Discussion

The results of the present work provide the first direct evidence for the involvement of IC adrenoceptors in cardiovascular responses observed during aversive threats. We have shown that bilateral microinjection of the selective α_1_-adrenoceptor antagonist WB4101 into the IC reduced restraint-evoked pressor and tachycardiac responses in a dose-dependent manner. Moreover, IC treatment with the selective α_2_-adrenoceptor antagonist RX821002 dose-dependently reduced MAP increase observed during restraint stress without affecting tachycardiac response.

Restraint stress is well accepted in the literature as an unconditioned and unavoidable aversive stimulus that elicits neuroendocrine and cardiovascular responses, the latter being characterized by sustained elevation of blood pressure, HR and the sympathetic activity that last through the restraint period [34]–[37]. The IC receives an organized representation of visceral information and is also highly interconnected with subcortical limbic and autonomic-related regions. Based on this combination of sensory input and limbic connectivity it has been descript as an important cortical center for the integration of autonomic and behavioral responses during aversive threats [13]. Conversely, we have demonstrated that CoCl_2_-induced acute bilateral inhibition of IC neurotransmission greatly attenuated both pressor and tachycardiac responses evoked by acute restraint stress [20]. However, due to the nonselective blockade of local neurotransmission caused by CoCl_2_ [21], [22], the possible neurotransmitter involved was not identified.

It has been showed that diverse array of physical (e.g., immune challenge, hypoglycemia, hypotension, and cold exposure) and emotional (e.g., immobilization, electric shock, loud noise, and restraint stress) stressors activate brain noradrenergic mechanisms [1], [23], [26], [38]–[42]. Noradrenergic neural terminals have been identified in the IC [30]. This IC innervation is mainly originated from noradrenergic cells grouped in the locus coeruleus (noradrenergic cell group A6) [27], [28], [30]. The present work has demonstrated that blockade of local α_1_-adrenoceptor by bilateral microinjection of WB4101 into the IC was able to reduce both pressor and tachycardiac responses evoked by restraint stress. These results corroborate with effects observed previously following CoCl_2_-induced acute bilateral inhibition of IC neurotransmission [20], thus suggesting that local α_1_-adrenoceptors mediates, at least in part, the IC influence on cardiovascular responses to restraint stress. Interestingly, blockade of local α_2_-adrenoceptors caused by microinjection of RX821002 into the IC also reduced restraint-evoked pressor response, but without affecting tachycardiac response. Therefore, present data suggest that control of cardiac function during restraint stress by IC noradrenergic neurotransmission is due a selective activation of local α_1_-adrenoceptors, whereas control of blood pressure during seems to be mediated by coactivation of local α_1_- and α_2_-adrenoceptors.

The presence of specific noradrenergic mechanisms within the IC controlling restraint-evoked pressor and tachycardiac responses indicates that different neuronal pathways originating in the IC are involved in control of vascular and cardiac functions during stress. The existence of specific central nervous system circuitries controlling autonomic activity to different organs provides the structural substrate for specific local IC noradrenergic neurotransmission mechanisms modulating cardiovascular adjustments during restraint [43]. Conversely, it has been demonstrated that several brain regions selectively modulate stress-evoked blood pressure and HR responses [44]–[47]. The presence of specific noradrenergic mechanisms in the central nervous system modulating vascular and cardiac responses to stress has also been reported [33], [48]. Therefore, our results corroborate with previous evidence of selective neural substrates controlling vascular and cardiac function during aversive threats.

Noradrenaline is released in several central nervous system regions, including the prefrontal cortex [23], [26], shortly after the onset of a stressful situation [1]. Since noradrenaline act through G protein-coupled receptors, which rapidly transfer their activation to downstream effectors, the rapid rise in their level is quickly translated into behavioral and physiological responses. This profile of fast release and action can explain why effects of local IC treatment with adrenoceptor antagonists are already observed during the early phase of restraint stress. However, it has been proposed that sustained and adaptive components of the stress responses (e.g., consolidation of the memory associated with the stressor) are mediated by mechanisms in the brain that affect gene expression and cell function [1]. A main mediator of these latter effects is corticosteroids acting through glucocorticoid receptors [1]. Since previous studies have demonstrated a role of the IC in the memory formation for aversive threat and hypothalamus-pituitary-adrenal axis control [2], [4], [9], further studies are necessary to investigate a possible role of IC in latter consequences of restraint stress.

Tachycardiac and pressor responses during stress are sympathetically mediated since they are abolished after the blockade of β- and α-adrenoceptors, respectively [5], [49], [50]. Moreover, treatment with parasympathetic blocker increases the tachycardiac response evoked by psychological stress [33], [51], [52], thus suggesting the simultaneous activation of cardiac parasympathetic and sympathetic activity during psychological stress. It has been reported that the IC modulate the sympathetic nervous activity through a mandatory synapse in the ventrolateral medulla [53], [54]. An IC control of cardiac parasympathetic activity has also been shown [31], [55]. Therefore, activation of IC α_1_-adrenoceptors could facilitate restraint-evoked tachycardiac response by stimulating facilitatory inputs to sympathetic medullary neurons and/or by stimulating inhibitory inputs to vagal neurons. Connections from the IC to sympathetic medullary neurons could also be the neural substrate for the facilitatory influence of IC α_1_- and α_2_-adrenoceptors on the pressor response to stress.

Baroreflex stimulus–response curve resets toward higher blood pressure values during aversive threat [5], [6]. It has been proposed that such changes on baroreflex activity play a facilitatory role in stress-evoked cardiovascular responses [3], [7]. We have previously demonstrated that IC noradrenergic neurotransmission acting through activation of α_1_-adrenoceptor modulates the baroreflex activity in a similar manner to that observed during stress [31]. Therefore, activation of IC α_1_-adrenoceptors could facilitate cardiovascular responses to restraint stress through its modulation of baroreflex activity. However, once IC treatment with selective α_2_-adrenoceptors does not affect baroreflex activity [31], it is possible that IC control of restraint-evoked pressor response through this adrenoceptor occurs by mechanisms independent of the baroreflex.

An antero-posterior organization of IC control of cardiovascular function has been proposed. Predominantly depressor responses have been reported following stimulation of rostral regions of the IC [13]. However, stimulation of the posterior IC elicits either pressor response associated with tachycardia (rostral sites within the posterior IC) or depressor response followed by bradycardia (caudal sites within the posterior IC) [13]. Although these pieces of evidence, a possible regionalization in the IC control of cardiovascular adjustments to stress has never been reported. The injection sites within the IC in studies investigating the role of this cortical region in the cardiovascular control during stress (including the present study) have centered within rostral regions of the IC [2], [20]. Therefore, further studies are necessary in order to investigate a possible rostro-caudal organization of the IC control of cardiovascular function during aversive threat.

IC treatment with adrenoceptor antagonists did not affect either MAP or HR baseline values. Therefore, although the present study supports the hypothesis that IC noradrenergic neurotransmission plays an important role in modulating the cardiovascular responses to restraint stress, this neurotransmission is not involved in the tonic maintenance of cardiovascular function. These results corroborate with our previous data demonstrating no changes in cardiovascular parameters after blockade of either glutamatergic receptors or adrenoceptors into the IC [16], [31]. However, present results contrast with data of other groups that observed increased arterial pressure and HR following microinjection of the neuronal blocker lidocaine into the IC of unanesthetized rats [53]. Lidocaine blocks both local synapses and passage fibers [56]. Therefore, since local IC pharmacological treatment with agents that selectively inhibits synapses without affecting passage fibers (e.g., CoCl_2_) does not affect cardiovascular basal parameters [20], it is possible that effects observed previously after local lidocaine treatment is due the inhibition of fibers passing through the IC and targeting other brain regions. Furthermore, it is important to mention that other studies did not identify effects of local lidocaine microinjection or IC lesion on cardiovascular baseline parameters [17], [18], thus supporting our results of absence of IC role in the tonic maintenance of cardiovascular function.

In conclusion, the present results show that noradrenergic neurotransmission in the IC modulates cardiovascular adjustments during restraint stress in a complex way. Our data provide evidence that IC noradrenergic neurotransmission acting through activation of both α_1_- and α_2_-adrenoceptors has a facilitatory influence on pressor response during acute restraint stress. Moreover, IC α_1_-adrenoceptors also play a facilitatory role on restraint-evoked tachycardiac response.
